# Alpha-Gal Syndrome Is More than Meats the IgE: A Patient Survey of Symptoms, Diagnosis, and Burdens

**DOI:** 10.3390/healthcare14162522

**Published:** 2026-08-13

**Authors:** Tina Merritt Meinholz, Kelly Cleary, Onyinye I. Iweala, Sarah K. McGill, Laura Rothfeldt, Anneke Walters, Melissa Olivadoti, Genevieve Weseman, Jennifer Platt

**Affiliations:** 1 Allergy and Asthma Clinic of Northwest Arkansas, Bentonville, AR 72712, USA; physician@allergyasthmaclinicnwa.com; 2 Food Allergy Research and Education (FARE), McLean, VA 22102, USA; kcleary@foodallergy.org; 3 Division of Rheumatology, Allergy, and Immunology, School of Medicine, University of North Carolina, Chapel Hill, NC 27599, USA; onyinye.iweala@med.unc.edu; 4 Division of Gastroenterology and Hepatology, School of Medicine, University of North Carolina, Chapel Hill, NC 27599, USA; mcgills@email.unc.edu; 5 Arkansas Department of Health, Little Rock, AR 72205, USA; laura.rothfeldt@arkansas.gov; 6 United Therapeutics Corporation, Xenotransplantation Research & Development, Blacksburg, VA 24060, USA; awalters@revivicor.com; 7 Assisi Consulting, LLC, Henderson, NV 89012, USA; 8 St. Charles County Department of Public Health, St. Charles, MO 63301, USA; 9 Tick Borne Conditions United, Pittsboro, NC 27312, USA; jennifer@tbcunited.org

**Keywords:** alpha-gal, alpha-gal syndrome, allergy, tick

## Abstract

**Highlights:**

**What are the main findings?**

- Our survey of patients with alpha-gal syndrome identified more systems affected after exposure to a variety of mammal products with a wider time frame than has previously been identified.
- Reactions occurred in all major body systems, and to a variety of products including meat, dairy, personal care products, and medications.

**What are the implications of the main findings?**

- Patients with alpha-gal syndrome may experience different types of symptoms in response to exposure that healthcare professionals should be aware of.
- More research needs to be done to fully characterize types of reactions to different products, and more education is needed to help healthcare professionals counsel patients on what products to avoid.

**Abstract:**

**Background/Objectives**: Alpha-gal syndrome (AGS) is an allergic condition that results in delayed symptoms in response to exposure to mammalian products. There is limited research on which healthcare professionals (HCPs) are diagnosing AGS, types of symptoms, time to reactions, and products that induce allergic responses in AGS. This survey study aimed to further identify the patient journey and characterize reactions for people with AGS. **Methods**: Adult patients with HCP-diagnosed AGS took a survey offered through social media and support organizations. Questions included diagnosing HCP type, time from exposure to symptom onset, and type of symptoms by body system. **Results**: Survey participants (n = 3437) were mostly white (95.8%) and female (81.5%), ranging from 18 to 86 years old, with a mean time from onset to diagnosis of 4.3 years. Most were diagnosed by an allergist (53.6%) or primary care doctor (30.7%). Participants reported gastrointestinal (86.2%), skin (83.5%), respiratory (59.8%), cardiovascular (41.3%), emotional (38.2%), motor (23.7%), and nervous system (23.3%) symptoms. Symptoms were most often reported at 4–6 h post-exposure (49.8%) and 6–8 h (28.6%), with a range from 0 min to 8+ hours. Products eliciting reactions included mammalian products (beef 97.7%, pork 87.2%, dairy 66.9%, gelatin 61.3%), prescription (41.3%) or over-the-counter medications (36.4%), and personal care products (42.0%). Most participants managed their reactions at home, and without epinephrine. **Conclusions**: This study demonstrates that patients with AGS may experience symptoms due to exposures from mammalian meat and a broad range of mammalian ingredient-containing products, including medications, personal care products, and dairy, with a wide range of body systems impacted. These results may be useful for educating patients and healthcare professionals to improve the accuracy and speed of AGS diagnosis.

## 1. Introduction

Alpha-gal syndrome (AGS) is an allergic condition most commonly associated with a tick bite, resulting in sensitization to the oligosaccharide galactose-α-1,3-galactose (alpha-gal) [1,2,3]. In the United States (U.S.), the Lone Star tick (*Amblyomma americanum*) was originally thought to be the only known vector after it was found in tick saliva [4]. However, additional ticks more recently implicated in the development of AGS in expanded territories include the Western black-legged tick (*Ixodes pacificus*) [5], the invasive Asian longhorned tick (*Haemaphysalis longicornis*) [6], the black-legged tick (*Ixodes scapularis*), and the American dog tick (*Dermacentor variabilis*) [4], expanding the areas of exposure. Vectors beyond ticks may also induce allergic sensitization to alpha-gal, including Trombiculidae (also known as chiggers) [5,7,8,9], intestinal helminths, and tsetse flies [10,11,12].

Affected individuals react to the alpha-gal carbohydrate found in a variety of mammalian meats and fats, as well as mammalian-derived products including dairy, gelatin, food additives, and ingredients in personal care items or household cleaning products [13,14]. Patients with AGS have also reported reacting to fumes emitted from cooking mammalian meat [13]. The concentration of alpha-gal found in non-primate mammals varies by species and tissue type, with organ meats of mammals containing the highest levels, which may cause responses to vary [15]. Research also now recognizes the presence of alpha-gal epitopes in other products such as the red algae carrageenan [13,16] and flounder roe [17].

As reactions can take hours to develop, AGS does not follow the same trajectory in age of onset, timing, or reaction types as other food allergies [18,19], which may lead patients and healthcare professionals (HCPs) to miss the symptoms of the allergy or patients to not recognize the connection between exposure and reactions.

The symptoms reported by AGS patients that have been documented include gastrointestinal, mucocutaneous, cardiovascular, and respiratory symptoms with an average of 2–6 h from exposure to reactions [13,19]. However, anecdotal reports have suggested that timing of reactions and types of symptoms can vary between and within patients, and patients may experience reactions in other body systems, which can complicate diagnosis. It is hypothesized that alpha-gal may be absorbed slowly into the circulation due to the amount of time it takes to absorb, process, and package glycolipids for transport throughout the body, which may be the reason for the delay and variation in symptoms that is often seen after alpha-gal exposure [4,20]. Yet, few studies have addressed the greater body system distribution, reaction type, and timing of symptoms in patients with AGS.

Currently published guidelines and expert opinion statements on AGS diagnosis vary on how best to identify AGS, rule out other conditions, and diagnose patients utilizing alpha-gal-specific IgE (sIgE) levels [13,21,22,23]. Recommendations often require a positive alpha-gal sIgE for diagnosis, but drops in serum levels of alpha-gal-specific IgE (sIgE) with the absence of subsequent tick bites, despite a persistence in reactions, have been documented [24]. Thus, some patients with AGS may have a negative alpha-gal sIgE after diagnosis and be told inappropriately that they are in remission, or may show negative testing upon presentation despite classic symptomatology, and may continue to go undiagnosed [13,21,22,23,25].

There is an absence of available preventative treatments to stop reactions to alpha-gal for those with AGS; therefore, the current management for patients diagnosed with AGS is avoidance of exposure to alpha-gal through diet, personal care products, and other carriers, along with avoidance of additional tick bites. Avoidance guidance documents and guidelines lack clarity and may make it difficult for HCPs to discuss the topics with patients, especially when medications and other products contain ingredients with hidden alpha-gal components such as (but not limited to) mammalian-derived magnesium stearate, lactose, or gelatin. Patients who do not have proper guidance or transparency in products may be at higher risk for exposure and resulting symptoms; however, there is no descriptive evidence to date on the types of products that result in symptoms, including the type and timing of symptoms.

There is also a significant lack of descriptive data on AGS symptomatology, time to response, alpha-gal sIgE levels, reported sources of exposure, how and when patients get diagnosed (i.e., the diagnostic pathway), and the treatments utilized in patients with AGS. We hypothesized that AGS has an extensive variety of bodily reactions, wider time frames for response, and a broader range of HCPs that diagnose AGS than previously reported.

To address this gap, we utilized a large-scale exploratory survey to characterize patient experiences with AGS, including patient characteristics, the diagnostic pathway, past and current exposure sources, timing of response, and types of reactions. Furthermore, this study sought to identify patterns in the experiences of patients with AGS that might lead to earlier AGS diagnosis and highlight key areas for future research and targeted HCP education.

## 2. Materials and Methods

### 2.1. Study Design and Participants

We conducted an open, voluntary, cross-sectional survey of individuals who self-identified as being diagnosed with AGS. Eligible participants were adults (≥18 years) who self-reported an HCP-confirmed AGS diagnosis and consented to the use of anonymized data. Portions of the survey were modeled after questions used in Food Allergy Research and Education’s (FARE^®^) food allergy registry [26]. Additional questions were included based on specific features unique to AGS. The survey data was deemed exempt by the University of North Carolina, Chapel Hill Institutional Review Board (IRB #24-1121). A pilot survey (n = 12) was conducted in November 2019 to assess instrument clarity and was adjusted to reflect participant feedback just prior to full roll-out of the survey in December 2019.

### 2.2. Data Collection

The full survey was administered via SurveyMonkey^®^ (San Mateo, CA, USA), and the link was disseminated through patient advocacy networks, nonprofit organizations, physician collaborators, and online support communities. All questions were optional except for respondent age and confirmation of AGS diagnosis by an HCP, which were required. For most questions, participants were required to choose one answer. Some questions had the option to choose multiple answer options (“check all that apply); analyses with questions with that option are noted below with the alpha (**^α^**) symbol. Data used for these analyses were collected between December 2019 and September 2024.

### 2.3. Data Processing

Survey responses were de-identified, and data was exported to Microsoft Excel^®^ version 11 (Microsoft Corporation, Redmond, WA, USA) and analyzed in R version 4.3.1 (R Foundation for Statistical Computing, Vienna, Austria). Preprocessing steps included removal of empty or redundant variables, retention of unique submissions, and cleaning of data to remove non-numeric entries where a number was required (e.g., words were used in a question about the participants’ alpha-gal sIgE level). Duplicate submissions by IP address identification were removed, and only unique submissions were retained. Quality checks included validation of year distributions and confirmation that required fields were populated before analyses were completed.

### 2.4. Statistical Analysis

All analyses were descriptive and pre-specified. Counts and percentages were computed for categorical variables such as demographics, diagnosing HCP type, mammalian product exposures, post-exposure time to reaction, symptoms and body system groupings, and medical interventions. For multi-selected items, percentages used the number of respondents as the denominator.

For continuous variables (e.g., age at first allergic reaction or alpha-gal sIgE), central tendency and spread were summarized and applied pre-specified binning (e.g., decade bins for ages; custom IgE bins including a focused ≤ 10 IU/mL view). Year-stratified checks included response totals by year and contingency tables. Analyses were restricted to records with non-missing values for the variable of interest; for selected variables, we explicitly recoded blank/NA to “NONE OF THE ABOVE” before tabulation.

### 2.5. Tabulation and Visualization

Tabular summaries were rendered with *kableExtra* and DT (via *htmlwidgets/htmltools*), with long tables presented in scrollable frames. Visualizations were produced with *ggplot2* and included counts by year, histogram distributions, and categorical profiles by demographic characteristics or survey responses.

## 3. Results

### 3.1. Participant Characteristics and Demographics

Most of the respondents self-identified their race as White (95.8%) and were female (81.5%), and participants ranged from 18 to 86 years of age. The mean age at diagnosis was 49.6 years (standard deviation (SD) = 13.84), and the mean first age of reaction was 45.3 years (SD = 15.96), demonstrating a 4.3-year difference in means from development to diagnosis. Allergists were the most commonly reported HCP to diagnose AGS (53.6%), but roughly a third of participants (30.7%) were diagnosed by an MD or DO in a primary care setting. Additional HCPs reported as diagnosing AGS in this population included nurse practitioners (6.7%), gastroenterologists (4.8%), and immunologists (2.0%) (Table 1).

Most participants (71.9%) reported that, after they became allergic, they did not become tolerant of mammalian ingredients. A small percentage of participants (7.1%) noted becoming tolerant of mammalian products, and 21.1% of participants were unsure if they had become tolerant.

### 3.2. Alpha-Gal-Specific IgE Testing Results and Products Leading to Reaction

More than one-third of participants (36%) reported an alpha-gal sIgE of 0.1 to 1.9 on their first test, and 0.8% reported they had a result of <0.1. The percentage of participants reporting their result of <0.1 on their last (latest) test increased to 2% (Table 1).

Mammalian meats, such as beef or pork, followed by dairy and gelatin, were the most common alpha-gal-containing products to elicit reactions, with 97.7% reporting reacting to beef, 87.4% reacting to pork, 66.9% reacting to dairy, and 61.3% reacting to gelatin (Table 2).

Participants also reported reactions to mammalian ingredients in personal care products (42.0%) such as tampons, cosmetics, shampoos, and lotions, as well as mammalian ingredients in prescription medications (41.3%) or over-the-counter medications (36.4%) (Table 2).

### 3.3. Time to Symptom Development and Settings of Alpha-Gal Exposure

Participants reported a variety of time frames for reactions ^α^, with the most common being 4–6 h (49.8%), followed by 6–8 h (28.6%) and 3–4 h (26.3%), with time frames for symptom development ranging from 0 to 5 min (12.6%) to up to 8 h or more (16.4%) after exposure (Table 2). The most common locations of exposure ^α^ were reported at home (64.7%, 2224/3436), at a restaurant/eating out (56.1%, 1926/3436), at work (29.1%, 1001/3436), or at school (4.7%, 160/3436).

Participants also reported a high incidence of reactions prior to diagnosis, with 40.2% reporting 15 or more reactions, 26.6% reporting 1 to 4 reactions, and 13.7% reporting 5–9 reactions.

Participants could choose more than one option for exposure time.

**Figure 1:**
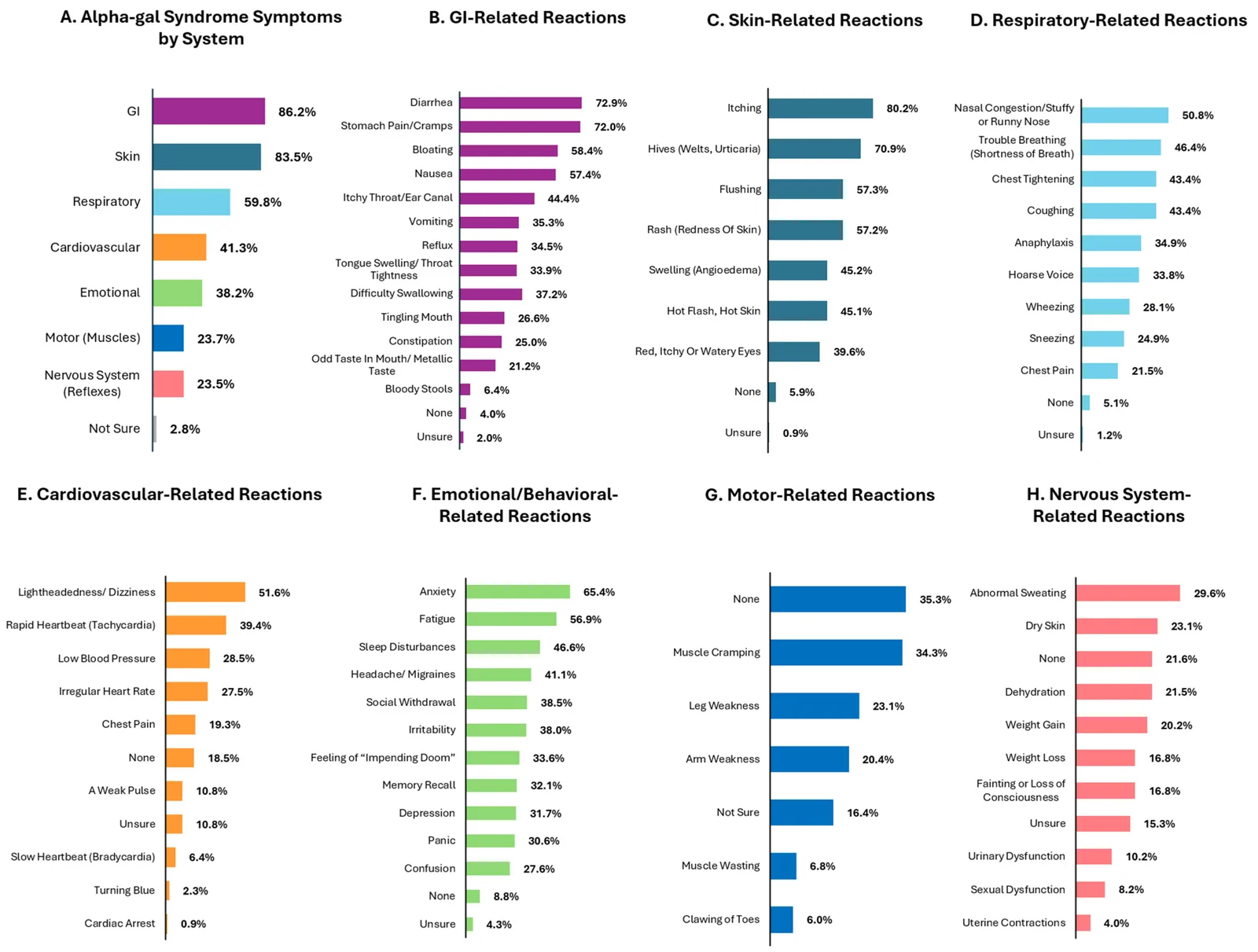
Reactions by body system. GI—gastrointestinal. N = 2594 for all graphs. All questions allowed for multiple symptom responses within each system.

Reactions were reported across all body systems, with the most common being gastrointestinal (86.2%) or skin manifestations (83.5%), followed by respiratory (59.8%) and cardiovascular symptoms (41.3%) (Figure 1A).

There was also high variability of symptoms within each body system (Figure 1B–H). More common symptoms included diarrhea, itching, rash, flushing, stomach cramps, bloating, nausea, anxiety, lightheadedness/dizziness, fatigue, nasal congestion, and trouble breathing.

Approximately half of participants (54.7%) reported managing symptoms at home/on their own; a total of 27.2% reported ever visiting the emergency room to manage a reaction, 7.48% reported ever visiting an urgent care facility, 4.6% reported ever self-administering epi-pen, 4.5% had been admitted to the hospital, and 1.6% had been admitted to the intensive care unit for AGS reactions.

### 3.4. Data Consistency

**Figure 2:**
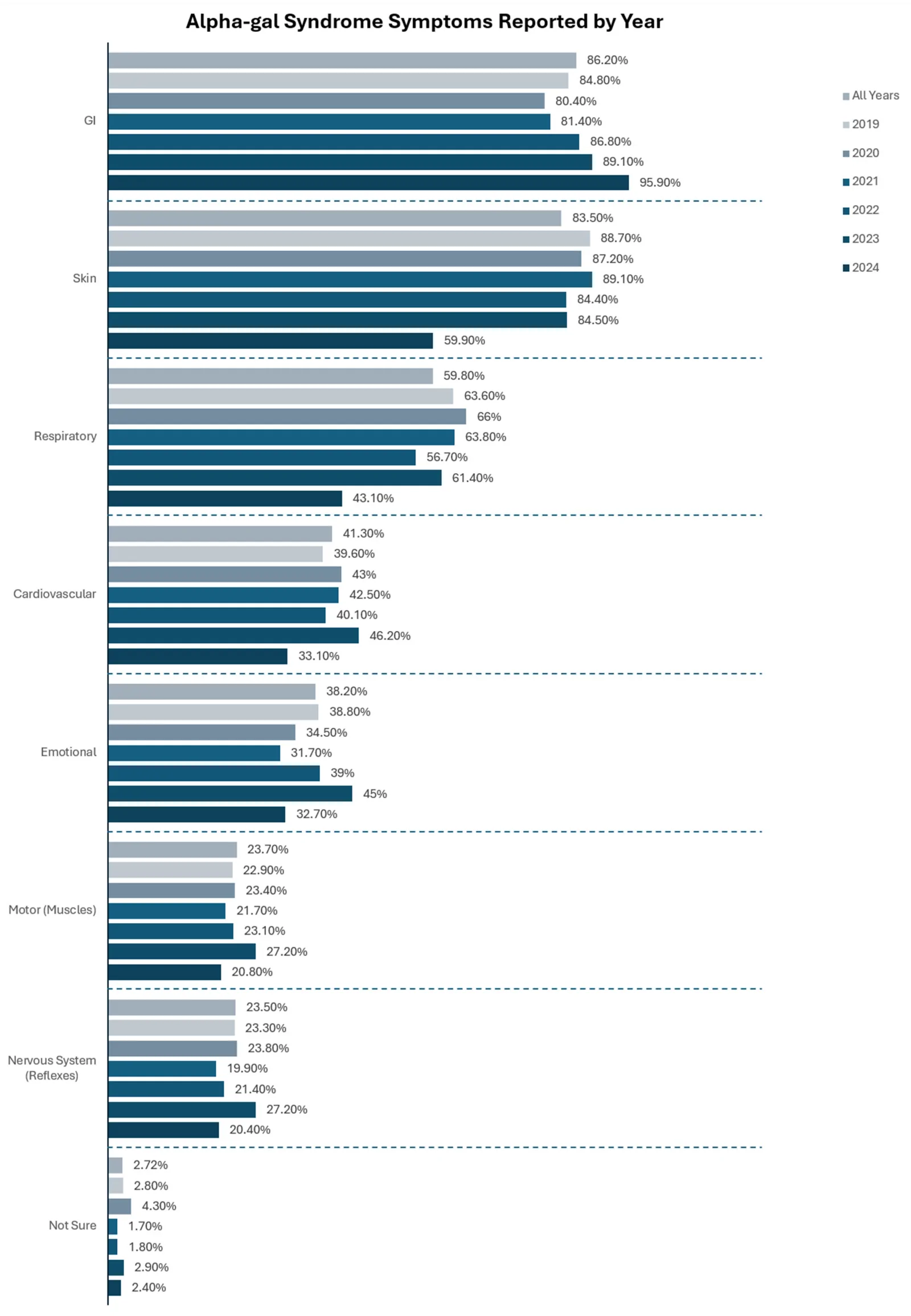
Percentage of alpha-gal symptoms reported by the year the survey was taken. GI—gastrointestinal.

Patient-reported data year-to-year during this multi-year survey are noted in Figure 2, along with the distribution between 2019 and completion in September of 2024 (for additional information including n’s, see Table S1). Statistics are descriptive, and thus it is not possible to identify significant differences or the potential reasons for distribution changes over time.

## 4. Discussion

Alpha-gal syndrome is a complex condition that can be difficult to diagnose, and even more difficult for patients to manage. Our survey results demonstrate that patients with AGS can experience a variety of symptoms across multiple body systems in response to mammalian products, and these reactions may not be readily recognized or correlated to exposure, or may be misdiagnosed as other conditions or allergies. Our finding of a mean 4-year delay in diagnosis from symptom onset is similar (albeit slightly shorter) to the 2017 findings by Flaherty et al. [27] of a mean of 7.1 years to diagnosis. It is plausible that patients taking the survey were more active in their interest in finding support and information on AGS, leading them to earlier diagnosis. In addition, our survey identified a larger constellation of symptom responses than previously reported [13,19]. Respondents also reported a high level of HCP and emergency room usage to manage reactions, consistent with findings from a previous smaller survey study [19]. This demonstrates a key need for better HCP education, earlier diagnosis, and easier management strategies for patients to improve time to diagnosis and decrease ER usage.

Mammalian meat was the most common product to elicit reactions, as has been frequently described [18,21,28,29,30]. Our study revealed that it was common to have reactions related to exposure at home and in restaurants. Cross-contact during manufacturing or cooking on shared surfaces can result in a reaction in both the home environment [10] and eating establishments, potentially impacting patients’ abilities and desires to eat at restaurants or use shared cooking surfaces in the home. Patients with AGS may also need to restrict activities related to food, or be required to disclose personal health information when explaining why they are not able to attend or eat at social gatherings. This, in turn, may impact social interactions and career development for patients with AGS, as has been previously reported for food allergies [31,32]. Future research on the quality of life and social impacts of AGS is needed.

This survey identified a high proportion of respondents with reactions following exposure to products beyond mammalian meat, including nearly two-thirds describing allergic responses to dairy, and more than a third describing responses to personal care products, prescription medications, or over-the-counter (OTC) medications. In the U.S., most personal care products and medications lack transparency concerning the origins of certain ingredients, as listing the source of specific ingredients is not required by the Food and Drug Administration (FDA). Both OTC and prescription drugs may contain mammalian-derived ingredients such as magnesium stearate (a common lubricant or flow agent used in medications) or lactose (a common binding agent), and manufacturing sources often change based on supply availability [33], making identification and avoidance of these products difficult.

Alpha-gal is also not required to be listed as an allergen on ingredient labels in the U.S., which is in stark contrast to requirements for transparency of ingredients in other allergen food-labeling standards. This leaves patients with AGS at risk of exposure to alpha-gal despite best efforts of avoidance. Modifying U.S. federal regulations on the labeling of food, personal care products, and medications to indicate the presence of alpha-gal, along with educating patients about potential sources, would help patients with AGS reduce accidental exposures.

Our results also demonstrate a broad range of times from alpha-gal exposure to reaction beyond the most commonly cited 2–6 h [20,34]. Patients could select more than one option for time to reaction post-exposure, and given the numbers of responses, participants likely experienced varied durations depending on the product they were exposed to and method of exposure. It is possible that earlier onset timing (reactions within minutes) could be due to direct systemic exposure of alpha-gal epitopes, such as through inhalation or injectable medications, or in products with higher amounts of the alpha-gal epitope [35]. Further studies are needed to understand the route and type of alpha-gal exposures, and how they relate to reaction time and symptoms, as the method of exposure (inhaled, ingested, injected, and skin contact), along with the type of product and amount of alpha-gal in the product, may affect responses, even within a single person. Further research is needed to identify patterns and connections between body systems and the attribution of symptoms to the appropriate system. As the question on post-exposure symptom expression allowed for multiple selections, it is possible that participants may have a range of reactions depending on the exposure route and type; future research is needed to identify symptom patterns and clustering.

While a positive alpha-gal sIgE is recommended by the CDC for diagnosis [13], 2% of participants who answered this question had a negative sIgE at their latest testing, which is a similar percentage found in other research [13]. Interestingly, this percentage increased from a report of 0.8% on their first test. This demonstrates that a subpopulation of AGS patients may be negative upon first testing, along with others becoming undetectable over time. Potential reasons for this could be due to decreases in serum levels of alpha-gal sIgE over time in the absence of tick bites and alpha-gal exposure [24], leading patients with a clinical history consistent with AGS to have negative alpha-gal sIgE upon presentation. It is also plausible that some patients achieved remission after showing undetectable sIgE levels. However, analyses to identify if those that achieved undetectable sIgE levels were due to full remission, a decrease in sIgE with symptoms present, or simply due to a lab error were not completed. These findings demonstrate that alpha-gal sIgE cannot be solely relied upon for diagnostic confirmation, and more sensitive and specific testing is needed to ensure patients receive appropriate diagnosis, remission, and treatment information in a timely manner.

Allergists and primary care practitioners were the most common HCPs identified as diagnosing AGS. Notably, dermatologists and gastroenterologists had a low percentage of diagnoses in this survey, despite high reports of responses related to the skin and gastrointestinal system, which demonstrates an important role that gastroenterologists and dermatologists can play in accelerating the time to AGS diagnosis with further education [21,30]. Specialists including allergists/immunologists, gastroenterologists, and dermatologists also need to have knowledge of AGS to properly differentiate it from other conditions that can present similarly, including irritable or inflammatory bowel syndrome, chronic urticaria, or mast cell activation disorder. Primary care practitioners are often the first stop for patients with allergic diseases [36], and may be relied upon for diagnosis if a diagnostic work-up and criteria are relatively straight-forward or access to a specialist is limited due to insurance coverage or availability.

Survey respondents identified symptoms and body systems following exposure to alpha-gal that have not been previously or commonly described, including neurological, psychiatric, neuroendocrine, respiratory, cardiovascular, mood, and motor impacts. Healthcare practitioners should be educated on the breadth and types of symptoms, testing limitations, and timing of reactions to be able to improve recognition and accurately diagnose and counsel patients with AGS.

Participants largely treated their reactions to alpha-gal while at home, which is not surprising, given that many reactions occur late at night or in the early morning after being exposed the previous day or evening [13]. Despite epinephrine being first-line treatment for AGS reactions [18], less than 5% of participants reported utilizing the treatment. This is consistent with other studies on the use of epinephrine in anaphylaxis for adults and pediatrics, due to out-of-pocket cost, lack of availability (i.e., not carrying their epinephrine), lack of symptoms’ relatedness or not seeing symptoms as severe enough, product expiration, or perceiving a need to go to the ER after usage [37,38,39,40,41]. Patient education on appropriate usage of injectable or intranasal epinephrine, along with improved access to these therapies, is needed for the appropriate treatment of AGS reactions.

Treatment of acute presentations frequently includes oral antihistamines or corticosteroids [42]. However, it is important to note these medications are not recommended for anaphylaxis [43]. Many antihistamine medications may have excipients or non-active ingredients that contain alpha-gal, including stearic or lactic acid, magnesium stearate, or lactose monohydrate, putting patients at further risk for subsequent or prolonged reactions. Drowsiness or loss of memory can also occur with recurrent first-generation antihistamine use [44,45]. Our data demonstrate that some patients may be seen at emergency rooms (ER) if reactions are severe, necessitating the education of ER HCPs about AGS presentations for effective treatment and counseling of patients.

This study has several limitations. As a real-world survey, the study relied on a participant’s self-attestation of diagnosis and did not require clinician attestation, review of medical records, or verification of sIgE testing to participate in the study, potentially leading to misclassification or recall bias. In addition, approximately 64% (n = 1503/2362) of participants gave a response to the question about sIgE levels. Symptoms were also self-reported, which means that results were contingent on participants’ understanding of relationships between reported symptoms and alpha-gal exposure, along with appropriate attribution to exposures, potentially introducing recall bias or misattribution. While the questions referred specifically to responses to items containing alpha-gal, it is possible that participants with co-occurring conditions that mimic AGS, such as other allergies or immunologic conditions, could have misattributed their symptoms to AGS. An aspect of the study that may limit generalizability to the overall population is utilizing a sample of convenience, which may have led to the lack of ethnic and sex diversity, with the population being largely white and female. In a military recruit blood sampling study, white individuals were more likely than Black and Hispanic participants to test positive [46], and a retrospective chart review study identified a higher incidence in females than males [47]; however, the true demographic distribution of AGS is unknown, so it is not possible to identify if our sample is representative of the AGS population.

Given the route of data collection (an easily accessible online survey), people who felt their reactions from AGS were not commonly recognized may have been more motivated to respond, skewing the results to less common presentations, those that had longer delays to diagnosis (resulting in a higher interest in awareness), or represent patients who are highly symptomatic, limiting generalizability to the larger population, potentially leading to self-selection bias. The survey also sourced participants from online support groups via social media; research has shown that women are more likely to seek social support when faced with challenges, which may have skewed our sample towards female respondents [48]. In addition, our sample likely included people who were actively seeking assistance through support groups or awareness organizations and therefore may pay closer attention to their condition. Due to these factors, this sample may not be fully representative of the AGS population. Further research expanding the demographics and sex of the sample, along with a secondary confirmation of diagnosis, is needed to fully apply these conclusions to the larger population. Currently, there is an absence of validated symptom and experience questionnaires for AGS. While the questions utilized from FARE’s food allergy registry were validated, additional questions were added to this survey in order to identify potential responses for further research and validation. However, this poses a risk for biased or unreliable data in our dataset that would require future validation and more robust research to make strong conclusions. The instrument included several questions where patients could choose more than one option (e.g., types of symptoms and time to reaction), which may make them more difficult to interpret. In addition, all responses were optional beyond the type of diagnosed HCP and age (≥18), leading to varying response numbers per question. As the large majority (58/60) of questions were optional, participants could have also experienced “survey fatigue” [49], potentially leading to questions towards the end of the survey receiving lower response rates.

## 5. Conclusions

Our survey demonstrates that clinical manifestations of AGS may present with variable symptoms across multiple body systems, in a larger range of post-exposure time frames from a variety of exposure sources. Given the heterogeneity in the presentation of patients with AGS and the limitations of alpha-gal sIgE diagnostic testing, further research and awareness of the depth and breadth of patient-reported experiences is essential for early identification. Integrating the patient perspective, including various sources of exposures, symptoms, body systems, and reaction times into future studies will be critical for more accurate characterization of symptomatology, timely diagnosis, and real-world management, as well as recognizing the impact on patients.

Due to the limitations of this real-world survey study and the potential for recall bias, self-reported diagnosis, self-selection bias, and the interpretation of non-food exposures, further research is needed that utilizes controlled studies with confirmed diagnosis, expanded demographics, and validated questions while requiring responses to all prompts to confirm these findings. This would include clarification of unique response times and symptom phenotypes in relation to product exposures, exploring the impacts of continued exposure to mammalian products (including dairy) even in asymptomatic situations, as well as psychological and quality of life impacts on patients. These data demonstrate that a broader range of symptoms and responses to alpha-gal should be considered for future research and potential inclusion in the diagnostic criteria for AGS, and that targeted education of HCPs and patients is needed to improve diagnosis and counseling for patients with AGS.

Given the potential for exposure to alpha-gal in products beyond mammalian meat and dairy, it is crucial for food, pharmaceutical, and manufacturing companies to list out mammalian products and excipients for consumer awareness to reduce exposures and impacts on patients with AGS. Finally, more work needs to be done to identify AGS in the absence of detectable alpha-gal sIgE, and to improve testing sensitivity and specificity to allow for earlier identification, diagnosis, and management of patients with AGS.

## Figures and Tables

**Table 1:** Participant characteristics and demographics *.

Characteristic/Demographic	Participants, n (%)
**Gender (n = 2312)**	
Male	427 (18.47)
Female	1885 (81.53)
**Race (n = 2269)**	
White	2174 (95.81)
American Indian/Alaskan Native	48 (2.12)
Unknown	21 (0.93)
Asian	10 (0.44)
Black/African American	9 (0.40)
Hawaiian/Pacific Islander	7 (0.31)
**Educational Level (n = 2257)**	
Bachelors	622 (27.56)
Some College	427 (18.92)
Masters	365 (16.17)
Associates	246 (10.86)
High school/equivalent	230 (10.19)
Trade/technical/vocational	172 (7.62)
Doctorate	73 (3.23)
Professional Degree	67 (2.97)
Some High School	56 (2.48)
**Age in years (n = 2275)**	
All patients; range	18–86
Age at diagnosis; mean (SD)	49.6 (13.84)
Age at first reaction; mean (SD) (n = 2370)	45.26 (15.96)
**HCP Diagnosis (By Type) (n = 2362)**	
Allergist	1227 (53.60)
Primary care (MD/DO)	702 (30.67)
Nurse practitioner	154 (6.73)
Gastroenterologist	109 (4.76)
Immunologist	45 (1.97)
Physician assistant (primary care)	36 (1.57)
Obstetrician/gynecologist	5 (0.22)
Cardiologist	3 (0.13)
Endocrinologist	3 (0.13)
Pediatrician	3 (0.13)
Dentist	2 (0.09)
**Alpha-gal IgE Result (First Test) (n = 1639)**	
<0.1	13 (0.80)
0.1–1.9	590 (36.00)
2.0–9.9	433 (26.40)
≥10.0	603 (36.90)
**Alpha-gal IgE Result (Last Test) (n = 1503)**	
<0.1	30 (2.00)
0.1–1.9	583 (38.80)
2.0–9.9	410 (27.30)
≥10.0	480 (31.90)
**Became Tolerant to Mammalian Ingredients (after having been allergic prior) (n = 2594)**	
No	1865 (71.90)
Unsure	546 (21.05)
Yes	183 (7.05)

* N’s and percentages shown include participants who answered the question; all questions beyond age and being diagnosed with AGS by an HCP were optional.

**Table 2:** Characteristics of reactions to alpha-gal-containing products.

Reaction Outcomes	
**Products that Elicit a Reaction (n = 2498) ^α^**	**Participants Reporting, n (%)**
Beef	2423 (97.70)
Pork	2163 (87.22)
Dairy	1656 (66.77)
Gelatin *	1519 (61.25)
Mammal ingredients in personal care products **	1042 (42.02)
Mammal ingredients in prescription medications	1024 (41.29)
Mammal ingredients in over-the-counter medications	903 (36.41)
Lamb	841 (33.91)
Mammal ingredients in household products ***	798 (32.18)
Venison	772 (31.13)
Mammal ingredients in vaccinations	384 (15.48)
Mammal ingredients in medical supplies	323 (13.02)
Mammal ingredients in textiles	309 (12.46)
**Post-Exposure Response Time (n = 2594) ^α^**	**Participants Reporting “Yes”, n (%)**
0–5 min	328 (12.64)
6–30 min	472 (18.20)
31–60 min	403 (15.54)
1–2 h (61–120 min)	569 (21.94)
2–3 h (120–179 min)	471 (18.16)
3–4 h (180–239 min)	681 (26.25)
4–6 h	1291 (49.77)
6–8 h	742 (28.60)
8 h or more	425 (16.38)
Unsure	159 (6.13)
**Incidence of Reaction Episodes Prior to Diagnosis (n = 2504)**	**Participants Reporting, n (%)**
15 or more	1007 (40.22)
10 to 14	140 (5.59)
5 to 9	343 (13.70)
1 to 4	667 (26.64)
Not Sure	310 (12.38)
None	37 (1.48)

* Reaction to either a food or ingredients in products (i.e., gelcaps, additives). ** Examples given—tampons, cosmetics, shampoo, and lotions. *** Examples given—cleaning products, laundry detergent, and dryer sheets. ^α^ Questions that had the option to “check all that apply” for answers. Sums of these percentages may be higher than 100%.

## Data Availability

The datasets presented in this article are not readily available because analyses were completed utilizing a larger dataset that has not been fully analyzed or published. Requests to access the datasets should be directed to Jennifer Platt at jennifer@tbcunited.org.

## Supplementary Materials

The following supporting information can be downloaded at: https://www.mdpi.com/article/10.3390/healthcare14162522/s1; Table S1: Alpha-gal symptoms by year.
