# Supplementary material for: Alpha-Gal Syndrome Is More than Meats the IgE: A Patient Survey of Symptoms, Diagnosis, and Burdens
Source: Healthcare (Basel). 2026 Aug 13;14(16):2522. doi: 10.3390/healthcare14162522 (PMC13511933; doi:10.3390/healthcare14162522)
Supplement: Supplementary file 1 [file healthcare-14-02522-s001.zip › healthcare-4406586-supplementary.pdf]

Table S1. Alpha-gal Symptoms by Year

Year	Total Responses to Question (Yes)	Total Responses by Year	Percentage Responded (Yes)
Gastrointestinal	2236	2591	86.3
2019	396	467	84.8
2020	378	470	80.4
2021	180	221	81.4
2022	507	584	86.8
2023	517	580	89.1
2024	258	269	95.9
Skin	2165	2591	83.6
2019	414	467	88.7
2020	410	470	87.2
2021	197	221	89.1
2022	493	584	84.4
2023	490	580	84.5
2024	161	269	59.9
Respiratory	1551	2591	59.9
2019	297	467	63.6
2020	310	470	66
2021	141	221	63.8
2022	331	584	56.7
2023	356	580	61.4
2024	116	269	43.1
Cardiovascular	1072	2591	41.4
2019	185	467	39.6
2020	202	470	43
2021	94	221	42.5
2022	234	584	40.1
2023	268	580	46.2
2024	89	269	33.1
Emotional	990	2591	38.2
2019	181	467	38.8
2020	162	470	34.5
2021	70	221	31.7
2022	228	584	39
2023	261	580	45
2024	88	269	32.7
Motor (Muscles)	614	2591	23.7
2019	107	467	22.9
2020	110	470	23.4
2021	48	221	21.7

2022	135	584	23.1
2023	158	580	27.2
2024	56	269	20.8
Nervous System (Reflexes)	603	2591	23.3
2019	109	467	23.3
2020	112	470	23.8
2021	44	221	19.9
2022	125	584	21.4
2023	158	580	27.2
2024	55	269	20.4

The numbers in grey rows indicate the total number across all years for participants who: reported symptoms in that system (“Total Responses to Question (Yes)”); the total number of participants who answered the question (“Total Responses by Year”); or the percentage of participants who reported symptoms in that system (“Percentage Responded (Yes)”).
